# How peer relationships affect academic achievement among junior high school students: The chain mediating roles of learning motivation and learning engagement

**DOI:** 10.1186/s40359-024-01780-z

**Published:** 2024-05-16

**Authors:** Yanhong Shao, Shumin Kang, Quan Lu, Chao Zhang, Ruoxi Li

**Affiliations:** 1 Jiangsu Xiangshui High School, Yancheng, China; 2 College of Foreign Languages, Qufu Normal University, Qufu, China; 3 College of Economics and Management, Tarim University, Alar, China; 4 Shandong Vocational Animal Science and Veterinary College, Weifang, China

**Keywords:** Peer relationship, Learning motivation, Learning engagement, Academic achievement, Junior high school students

## Abstract

**Background:**

Despite the recognition of the impact of peer relationships, learning motivation, and learning engagement on academic achievement, there is still a gap in understanding the specific mechanisms through which peer relationships impact academic achievement via learning motivation and learning engagement.

**Methods:**

This study aims to investigate how peer relationships affect junior high school students’ academic achievement through the chain mediating roles of learning motivation and learning engagement, employing the self-system model of motivational development as the theoretical framework. In January 2024, 717 participants were selected from two middle schools in eastern China (mean age = 13.49 years, SD = 0.5). The data analysis in this study was performed using the structural equation model (SEM) in AMOS 24.0 and SPSS 24.0.

**Results:**

The results showed that peer relationships were directly and significantly related to junior high school students’ academic achievement, and that peer relationships were indirectly and positively related to junior high school students’ academic achievement via learning motivation and learning engagement respectively. The results also revealed a significant indirect and positive relationship between peer relationships and junior high school students’ academic achievement, mediated by the sequential mediating roles of learning motivation and learning engagement. Moreover, the path “peer relationship→learning motivation→academic achievement” has the strongest indirect effect.

**Conclusion:**

For junior high school students to achieve academic success, the appropriate interventions should be implemented to improve peer relationships, learning motivation, and learning engagement.

## Introduction

Academic achievement is a multifaceted construct that can be defined in broad and narrow aspects. Marsh and McCallum defined it broadly as the extent to which students achieve the objectives or goals of their educational institution or program [1]. In contrast, Hattie defined it narrowly as the progress that students make in their academic studies, demonstrated through their performance on tests, exams, and other assessments [2]. Many researchers have adopted the narrow definition, focusing on test scores in specific subjects [3–5]. In China, academic achievement is often measured by test scores in Chinese, Math, and English [6, 7]. Therefore, academic achievement in this study refers to students’ test scores in these subjects. Academic achievement holds substantial importance not only for students’ future prospects but also serves as a critical indicator for evaluating the effectiveness of national educational systems [8].

Peer relationships have been recognized as influential factors in adolescents’ academic achievement [9]. Peer relationships refer to the social interactions and connections that individuals establish with their peers, including interpersonal relationship, social emotion, communication interaction [10]. They can have a profound impact on students’ academic outcomes, as peers can serve as sources of both positive and negative influence. Positive peer relationships have been associated with higher levels of academic achievement, while negative peer relationships can hinder students’ academic progress [11]. 

Learning motivation and learning engagement are two psychological constructs that have been extensively studied in relation to academic achievement [12]. Learning motivation encompasses the internal drive and inclination to participate in learning activities, which can be classified into two main categories: intrinsic motivation and extrinsic motivation [13]. Intrinsic motivation stems from personal interest, curiosity, and the inherent satisfaction derived from the learning process itself, while extrinsic motivation is driven by external factors such as rewards, grades, or social recognition [14]. Learning engagement encompasses the active involvement, effort, and persistence that individuals exhibit during the learning process, categorized into three components: vigor, dedication, and absorption [15]. Vigor is often used to describe an individual’s level of enthusiasm, engagement, and persistence in their studies. Dedication refers to an individual’s commitment and devotion to their academic pursuits. Absorption refers to an individual’s deep focus and concentration on what is studied [16]. Both learning motivation and learning engagement have been found to exhibit a positive correlation with academic achievement. For example, Wentzel suggested that learning motivation plays a positive role in academic achievement [17]. Similarly, Li et al. observed a noteworthy positive association between academic motivation and mathematics achievement among junior high school students [18]. Liem and Martin posited that school engagement has a positive impact on academic performance [19]. The findings highlight the importance of considering both learning motivation and learning engagement in understanding academic achievement.

Despite scholars proposing the influence of these factors on academic achievement, the specific mechanisms through which peer relationships influence academic achievement via learning motivation and learning engagement remain underexplored. To address this research gap, the primary objective of the current study is to investigate the interactive effects of peer relationships, learning motivation, and learning engagement on academic achievement, thereby providing a holistic comprehension of the interplay between these factors. Furthermore, this study endeavors to examine the chain mediating roles of learning motivation and learning engagement in the association between peer relationships and academic achievement among junior high school students. By examining these mediating pathways, this study seeks to elucidate the underlying mechanisms by which peer relationships impact academic outcomes. This study differs from those in investigating the chain mediating roles of learning motivation and learning engagement in the association between peer relationships and academic achievement within a unified conceptual framework, contributing to a deeper understanding of the factors that shape students’ academic success.

**Fig. 1:**
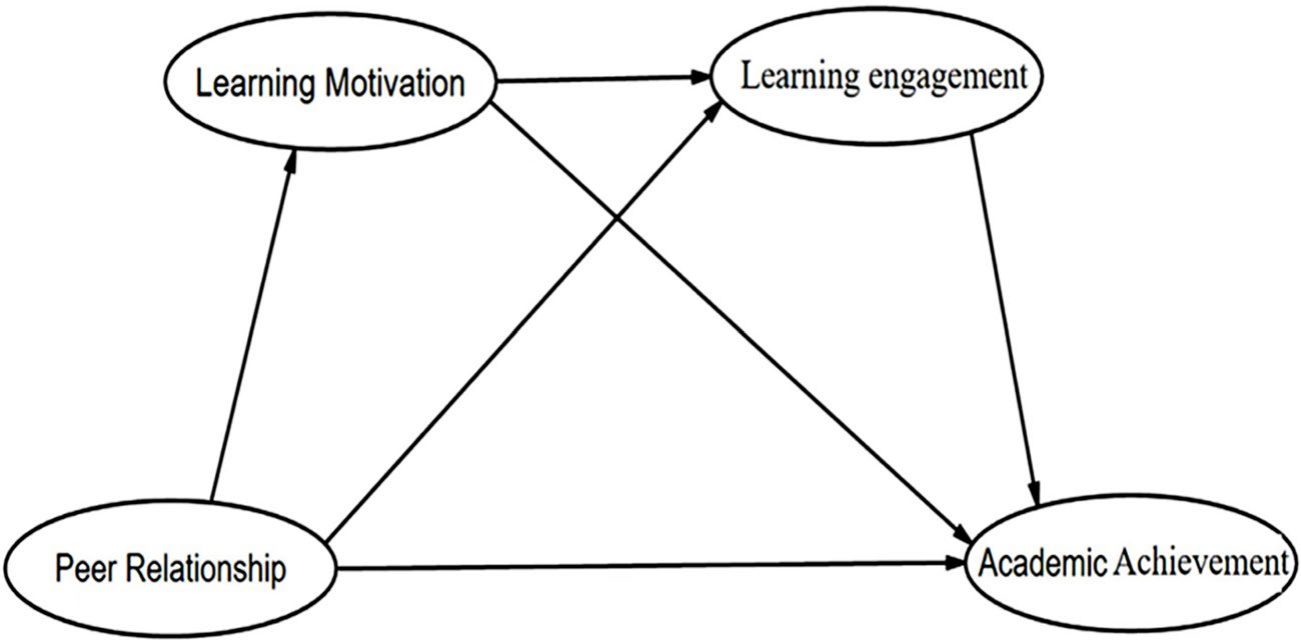
The proposed theoretical model

The self-system model of motivational development (SSMMD) serves as a conceptual framework for this study. Proposed by Connell and Wellborn [20] and supported by Skinner et al. [21], the SSMMD is rooted in the self-determination theory [22] and emphasizes the importance of individuals’ intrinsic motivation and psychological needs for autonomy, competence, and relatedness [23]. The SSMMD comprises four interconnected components: social context, self-system, action, and developmental results. The social context, consisting of peers, teachers, and parents, shapes an individual’s self-system. It is within this social context that an individual’s self-beliefs, motivation, and engagement in activities are developed. The self-system, as a relatively stable personal resource, is influenced by long-term interactions with the surrounding context and can effectively predict the level of involvement in activities. This level of involvement, in turn, directly influences various aspects of an individual’s development, including behavior and academic performance [24]. The SSMMD presents a linear developmental pathway, where the social context influences the self-system, which then influences actions and subsequently developmental outcomes. In this study, we utilize the SSMMD framework to explore the relationship between peer relationships, learning motivation, learning engagement, and academic achievement. The relationship between the four variables and SSMMD can be elaborated as follows: Peer relationships, as a component of the social context, shapes an individual’s self-beliefs, which significantly influences their learning motivation. Students who possess higher levels of learning motivation are more likely to get active engagement in learning activities (as a component of the action), and impact their academic achievement positively (as a developmental outcome) [25]. Based on this model, this study hypothesizes that peer relationships (as a social context factor) may influence adolescents’ learning motivation (as a self-system factor), which in turn affects their learning engagement (as individual action), ultimately resulting in a positive impact on academic achievement (as developmental outcomes). This theoretical model in the study is visually represented in Fig. 1.

### Peer relationships and academic achievement

Previous research has consistently demonstrated the positive influence of peer relationships on academic achievement [26]. Several studies have examined the positive impact of peer relationships on overall academic achievement. For instance, Wentzel noted that peers’ support in homework was positively related to academic achievement [17]. Jacobson and Burdsal found that positive peer influence in middle schools predicted higher academic achievement [27]. In a longitudinal study, Gallardo et al. (2016) demonstrated the positive influence of peer relationships on mid-adolescents’ academic achievement [11]. Additionally, research has investigated the positive effects of peer relationships on academic achievement in specific subjects. For example, Li et al. reported a significantly positive effect of peer relationships on the mathematics achievement of junior high school students [18]. Li et al. (2020) identified a significantly positive connection between peer relationships and science literacy among 596 ethnic minority junior school students in China [28]. Moreover, previous studies have suggested that the positive impact of peer relationships on academic achievement increases with grade level [29] and that same-gender peer relationships are particularly important in predicting academic achievement [19]. Overall, these findings emphasize the critical role of positive peer relationships in academic achievement, highlighting that adolescents who cultivate supportive relationships with their peers are more inclined to achieve success in their academic pursuits. On the basis of this, the following hypothesis is proposed.

H1: Peer relationships are positively correlated with academic achievement.

### Learning motivation as a mediator

Peer relationships have been demonstrated to have a significant influence on learning motivation [11]. Positive peer relationships can enhance students’ motivation in learning by providing support, encouragement, and a sense of belonging. For example, Li et al. have indicated that positive peer relationships could encourage students to strive towards predetermined learning goals [30]. Similarly, Kuo et al. have shown that regular peer interaction could increase students’ motivation and interest in learning [31]. Wentzel et al. conducted a questionnaire survey involving 240 participants, and found that adolescents who receive positive support from their peers are more prone to exhibit higher levels of motivation [32]. In a study by Huangfu et al. it was observed that peer support in the context of chemistry education had a significant positive impact on students’ continuing motivation in chemistry [33]. Conversely, negative peer relationships can lead to decreased motivation. For instance, Juvonen and Graham found that students who experienced bullying, as a form of negative peer relationship, reported lower levels of motivation to engage in academic tasks [34]. Similarly, Wentzel et al. revealed that peer rejection, as another form of negative peer relationship, was associated with lower levels of intrinsic motivation in students [35]. These finding underscore the crucial role of peer relationships in influencing students’ motivation in specific academic domains.

Furthermore, learning motivation has been found to have a positive correlation with academic achievement [36]. Students who possess high levels of motivation to learn tend to excel in classroom activities, put forth great effort to complete their learning assignments, and achieve their academic achievement [37]. Researchers have demonstrated that learning motivation, as a potential mechanism is associated with perceived academic achievement [38]. Moreover, intrinsic motivation has been found to have a positive correlation with students’ grades, while extrinsic motivation shows a negative association with academic outcomes [39]. In addition, researchers have shown that learning motivation exerts both direct and indirect influences on students’ academic achievement through learning activities [40]. Peer interactions have also been emphasized as influential factors in adolescent learning motivation and subsequent learning outcomes [41]. Li et al. highlighted the mediating role of learning motivation in the relationship between peer relationships and mathematics achievement [18]. Although the study focused on Zhuang ethnic minority students in China and limited the academic achievement to mathematics, it provides valuable insights and direction for the mediation hypothesis in this research. Based on these findings, the following assumptions are proposed:

H2: Peer relationships are positively correlated with learning motivation.

H3: Learning motivation is positively correlated with academic achievement.

H4: Learning motivation mediates the association between peer relationships and junior high school students’ academic achievement.

### Learning engagement as a mediator

Research has consistently shown that peer relationships have an impact on students’ learning engagement [42]. For instance, Kiefer et al. have proposed that peer support may help middle school students improve their learning engagement [43]. Besides, Research has demonstrated that both academic and emotional support from peers can enhance students’ learning engagement [44]. Lee et al. have claimed that peer interaction can help students sustain their engagement in e-learning [45]. In addition, Yuan and Kim have suggested that peer appraisal in peer interactions can affect teenagers’ cognitive and emotional involvement [46].

Learning engagement is considered to be an important factor that affects students’ academic achievement [12]. High levels of learning engagement allow students to devote more time to learning activities and ultimately achieve better academic outcomes [47]. Liem and Martin found that active participation and investment in learning activities positively predict academic success [19]. Wang et al. further supported this by demonstrating that higher levels of classroom engagement are associated with better academic performance [4]. Additionally, Saqr et al. highlighted the longitudinal effects of engagement, showing that sustained high levels of engagement lead to improved academic outcomes over time [48]. Taken together, these recent studies underscore the critical role of student engagement in fostering academic achievement.

Learning motivation has been demonstrated to have a significant impact on students’ engagement in learning activities [49]. When students are motivated to learn, they are more likely to set ambitious goals and actively participate in their learning activities [50]. Research has consistently found a positive relationship between learning motivation and engagement [25, 41]. For instance, a study by Froiland and Worrell explored the role of motivation in student engagement and found that intrinsic motivation, which stems from personal interest and enjoyment, was positively associated with higher levels of engagement [51]. Similarly, a study by Huang and Yang highlighted the importance of learning motivation, where students feel a sense of desire and enjoyment in their learning, in promoting engagement [52]. The self-system model of motivational development suggests that social contexts, including interactions with peers, can impact students’ self-systems, such as their motivation and self-efficacy in learning. When students’ self-systems, including learning motivation, are strengthened, they are more likely to engage in learning activities, leading to improved academic outcomes, such as academic achievement. Therefore, based on the aforementioned research, it is postulated that peer relationships can promote academic achievement by enhancing students’ motivation and engagement in learning activities. Hypotheses were derived from the aforesaid analysis:

H5: Peer relationships are positively correlated with learning engagement.

H6: Learning motivation is positively correlated with learning engagement.

H7: Learning engagement is positively correlated with academic achievement.

H8: Learning engagement mediates the association between peer relationships and junior high school students’ academic achievement.

H9: Learning motivation and learning engagement play a chain mediating role in the association between peer relationships and junior high school students’ academic achievement.

## Materials and methods

### Sampling and data collection

Prior to conducting the survey, ethical approval and support were obtained from the Ethics Committee of Qufu Normal University. To ensure the privacy and confidentiality of the students, several measures were implemented. Firstly, the personal identification information of the students was anonymized, with the utilization of student ID numbers instead of real names on the questionnaire. Secondly, explicit assurances were provided to the participants that designated members of the research team would have access to and process the collected data. Lastly, strict adherence to legal regulations and ethical guidelines was maintained throughout the entire research process.

The sample size for the study was determined based on the guidelines of Structural Equation Modeling (SEM), which recommend a sample size of at least ten times the total number of observed variables [53]. Consistent with this recommendation, a sample of 717 participants, aged 13–14 years old, was drawn from two middle schools in Jiangsu province, Eastern China, in January 2024. The two schools selected for this study, in that they exhibit diversity in terms of student backgrounds, academic performance, and socio-economic status, reflecting the overall characteristics of students in the region. The participants were randomly chosen from Grades 7 and 8.

Data collection consisted of two distinct steps. Firstly, paper questionnaires were distributed with an explanation of the study. Students were encouraged to participate in the study voluntarily and express their ideas freely. Those who did not provide informed consent or failed to complete the questionnaire were excluded from the analysis. Totally, 717 valid questionnaires were collected, with a response rate of 89.6%. Secondly, the students’ academic achievement was also collected as part of the study. Specifically, the study collected scores from the final exams in the subjects of Chinese, math, and English as a measure of participants’ academic achievement, and matched the students’ grades with their IDs. To ensure comparability and facilitate analysis across different subjects, the overall scores, ranging from 0 to 120 were standardized. These standardized scores were then utilized as the observational variables of academic achievement.

### Research instruments

#### Peer relationship scale

Peer relationships were measured by the Peer Relationship Scale developed by Wei [10]. This scale comprises 20 items, categorized into three dimensions: interpersonal relationship (e.g., “My classmates all enjoy being with me.”), social emotions (e.g., “When I am with my classmates, I feel very happy.”), communication interaction (e.g., “If I see my classmates feeling upset or crying, I will go comfort them.”). The 5-point Likert scale was used, with scores ranging from 1 to 5 indicating “strongly disagree” to “strongly agree”, with higher scores indicating higher peer relationships. The scale has good reliability and validity, which has been validated by recent research [54].

### Learning motivation scale

Learning motivation was measured by the Learning Motivation Scale, developed by Amabile et al. [55], and later revised by Chi et al. [56]. This scale comprises 30 items, including two subscales for intrinsic motivation (e.g., “I enjoy independently thinking to solve difficult problems.”) and extrinsic motivation (e.g., “I care a lot about how others react to my opinions.”). The scale uses a 4-point rating, with scores ranging from 1 to 4, representing “strongly disagree” to “strongly agree”. Studies have demonstrated good reliability and validity of this scale among Chinese adolescents [49].

#### Learning engagement scale

Learning engagement was assessed by the scale revised by Fang et al. [57] based on the Utrecht Work Engagement Scale-Student (UWES-S) [58]. This scale comprises 17 items, including three dimensions: vigor (e.g., “I feel energized when studying.”), dedication (e.g., “When I study, I feel time flying.”), and absorption (e.g., “I take pride in my learning.”). The scale uses a 7-point rating, with scores ranging from 1 to 7, representing “Never” to “Always”. The scale demonstrated good reliability, which has been validated by An et al. [49]

### Academic achievement

Based on previous research [4–7], this study employed the final exam scores in Chinese, Mathematics, and English for grades 7 and 8 during the first semester as measures of academic achievement. A significant correlation was observed among the scores of these three subjects. Subsequently, the scores for each subject were standardized, and the average of these standardized scores was calculated as the overall indicator of academic achievement.

### Statistical analysis

Data analysis was conducted using Amos 24.0 and SPSS 24.0. Initially, the Harman single-factor test was performed to explore the possibility of common method bias. Subsequently, descriptive analysis was carried out to provide an accurate portrayal of the sample’s characteristics. Then, a structural equation modeling (SEM) analysis was conducted to test both the measurement and structural models. The measurement model was assessed through confirmatory factor analysis, while the structural model was evaluated by analyzing goodness-of-fit indices and path coefficients. Lastly, the significance of mediating effects was determined using the bootstrapping approach.

## Results

### Common method variance

To mitigate potential bias inherent in self-reported data obtained from junior high school students, the Harman single-factor test was conducted using SPSS 24.0 [59]. According to the test result, 11 factors exhibited characteristic roots exceeding 1, with the first factor accounting for 31.029% of the total variance, which fell below the critical threshold of 40% [60]. These findings suggest that no significant common method variance was present, indicating that the study’s reliability and validity were not substantially impacted.

### Sample characteristics

The sample was composed of 717 participants selected from two middle schools in eastern China. The average age of participants was 13.49 years (SD = 0.5, range = 13–14 years). As indicated in Table 1, the sample was gender-balanced, with males accounting for 50.1% and females accounting for 49.9%. The distribution of students across different grades was as follows: 53.7% in Grade Seven and 46.3% in Grade Eight. The majority of students resided in towns. Regarding the educational level of the participants’ fathers, 48.8% had completed junior high school or below, 36.8% had attended senior high school or vocational school, 8.9% had attended college, and 5.4% had attended university. Similarly, for the participants’ mothers, 51.9% had completed junior high school or below, 33.8% had attended senior high school or vocational school, 9.2% had graduated from colleges, and 5.2% had attended university.

**Table 1:** Descriptive overview of sociodemographic characteristics of the students

Demographic	Sample(*n* = 250)	Frequency	Percentage
Gender	Male	359	50.1%
	Female	358	49.9%
Grade	Grade Seven	385	53.7%
	Grade Eight	332	46.3%
Resident	Town	476	66.4%
	Countryside	241	33.6%
Fathers’ educational level	Junior high school or below	350	48.8%
	Senior high school or vocational school	264	36.8%
	College	64	8.9%
	university	39	5.4%
	Junior high school or below	372	51.9%
Fathers’ educational level	Senior high school or vocational school	242	33.8%
	College	66	9.2%
	university	37	5.2%

### Measurement model

The conventional approach to assessing a measurement model involves examining its reliability and validity [53]. In this study, the skewness of the 4 variables ranged from − 1.867 to 1.111, and the kurtosis ranged from − 0.351 to 3.512, which conforms to the normal distribution standards proposed by Hair et al. [61], providing a basis for the subsequent analysis. Reliability is commonly evaluated using Cronbach’s alpha, with coefficients from 0.80 to 0.89 considered acceptable. Convergent validity is evaluated through standardized factor loadings, composite reliability (CR), and average variance extracted (AVE), where values exceeding 0.5 are deemed acceptable [62]. Discriminant validity is assessed by comparing the square root value of AVE with the correlation coefficient value between constructs. It is generally expected that the square root value of AVE will exceed the correlation coefficient value [63]. 

Table 2 presents the results of the reliability and convergent validity analysis. The measurement model demonstrated acceptable reliability, as indicated by Cronbach’s alpha coefficients ranging from 0.839 to 0.961. Additionally, the standardized factor loadings ranged from 0.762 to 0.922, while the composite reliability (CR) and average variance extracted (AVE) values ranged from 0.835 to 0.937 and from 0.678 to 0.832, respectively, indicating acceptable convergent validity. Table 3 shows that the square root values of AVE for each construct were larger than the correlation coefficient values between the other constructs, indicating acceptable discriminant validity.

**Table 2:** Evaluation of reliability and validity

Latent variable	Dimension	SC	Cronbach’s a	CR	AVE
Peer relationship (PR)	IR	0.772–0.873	0.922	0.926	0.678
	SE	0.691–0.913	0.913	0.916	0.689
	CI	0.595–0.871	0.929	0.928	0.591
Learning motivation(LM)	IM	0.549–0.909	0.936	0.947	0.566
	EM	0.548–0.906	0.944	0.945	0.524
Learning engagement(LE)	VG	0.638–0.859	0.846	0.849	0.589
	DD	0.569–0.909	0.940	0.942	0.675
	AP	0.635–0.809	0.885	0.887	0.614
Peer relationship (PR)		0.907–0.915	0.961	0.937	0.832
Learning motivation(LM)		0.775–0.894	0.961	0.835	0.718
Learning engagement(LE)		0.862–0.915	0.946	0.862	0.678
Academic achievement(AA)		0.762–0.922	0.839	0.896	0.743

SC = standardized coefficients; IR = interpersonal relationship; SE = social emotion; CI = communication interaction; IM = intrinsic motivation; EM = extrinsic motivation; VG = vigor; DD = dedication; AP = absorption

**Table 3:** The test for discriminant validity of potential variables

Potential variable	Peer relationship	Learning motivation	Learning engagement	Academic achievement
Peer relationship	**0.912**			
Learning motivation	0.534	**0.847**		
Learning engagement	0.303	0.322	**0.823**	
Academic achievement	0.340	0.346	0.329	**0.862**

*Note* The square root of the AVE of four latent constructs is given in the diagonal, and the correlation coefficient is given on the below diagonal

### Structural model

The structural model was evaluated using the goodness-of-fit indices and path coefficients. Jackson et al. have suggested that a structural model fits the data when the goodness-of-fit index is between 1 and 3 for x^2^ / df, greater than 0.9 for GFI, AGFI, NFI, TLI, and CFI, less than 0.08 for SMSEA [64]. Table 4 displays the following fit indices: X^2^ / df = 1.142 (X^2^ = 2663.1543, df = 2331), GFI = 0.946, AGFI = 0.942, CFI = 0.993, TII = 0.993, NFI = 0.946. All the values met the recommended thresholds, indicating a good fit for the structural model. Additionally, sensitivity analysis indicated that the effect size was 0.49, meeting the threshold proposed by Cohen [65] for a strong statistical test with a sample size of 717.

**Table 4:** Goodness-of-fit indices for the structural model

Fit index	Suggested values	Value of this study
CMIN/DF(χ2/df)	>1 & <3	χ2 = 2663.1543, df = 2331, χ2/df = 1.142
Root mean square error of approximation (RMSEA)	< 0.08	0.014
Goodness of Fit Index (GFI)	> 0.90	0.946
Adjusted Goodness of Fit Index (AGFI)	> 0.90	0.942
Incremental Fit Index (NFI)	> 0.90	0.946
Comparative Fit Index (CFI)	> 0.90	0.993
Tucker-Lewis index (TLI)	> 0.90	0.993

### Hypothesis test

As depicted in Table 5, the results revealed a significant and positive association between peer relationships and academic achievement (β = *0.178*, *P* < 0.001), providing support for H1. A significant and positive correlation was observed between peer relationships and learning motivation (β = *0.534*, *P* < 0.001 ), conforming H2. Learning motivation was found to have a significant and positive impact on academic achievement (β = *0.181,P* < *0.001* ), thus supporting H3. Peer relationships exhibited a significant and positive influence on learning engagement (β = *0.183*, *P* < 0.001 ), providing support for H5. Learning motivation had a significant and positive effect on learning engagement (β = *0.224*, *P* < 0.001 ), thus H6 was supported. Learning engagement demonstrated a significant and positive impact on academic achievement (β = *0.217*, *P* < 0.001 ), providing support for hypothesis H7. Overall, the empirical data supported the expected directions of H1, H2, H3, H5, H6, and H7, indicating the significance of these relationships.

**Table 5:** The test results of path relationship

Hypothesis	Path	Unstand estimates	t	Sig.	Stand estimates	Hypothesis test
H1	PR→AA	1.313	3.712	***	0.178	Supported
H2	PR→LM	0.318	12.232	***	0.534	Supported
H3	LM→AA	2.239	3.512	***	0.181	Supported
H5	PR→LE	0.134	3.545	***	0.183	Supported
H6	LM→ LE	0.274	4.033	***	0.224	Supported
H7	LE→AA	2.192	4.875	***	0.217	Supported

*Note*: PR = Peer Relationship, AA = academic achievement, LM = Learning Motivation, LE = learning engagement

### Analyses of the mediating effect of peer relationship on academic achievement

In this study, Structural Equation Modeling (SEM) was employed as the statistical technique to examine the mediating effect of learning motivation and learning engagement. SEM is considered more appropriate for examining mediation [66]. To determine the confidence intervals for the mediation effects in SEM, the bootstrap method was utilized [67]. Specifically, a mediating effect is considered statistically significant when the 95% bias-corrected confidence intervals (95% bias-corrected CI)does not include 0, and t exceeds 1.96 [68]. For data analysis, Amos 24.0 software was utilized. In this analysis, academic accomplishment was considered as the dependent variable, while peer relationship was treated as the independent variable. Additionally, learning motivation and learning engagement were regarded as mediating variables. To enhance the reliability of our results, a bootstrap resample size of 5000 was utilized, and the bias-corrected confidence interval level was set at 95%.

The results indicated in Table 6 demonstrate the statistical significance of the total effect and direct effect of peer relationships on academic achievement. The total effect of peer relationships on academic achievement was 2.510 (t = 6.213, 95% bias-corrected CI [1.745, 3.309], *P* < 0.01), while the direct effect was 1.313 (t = 3.712, 95% bias-corrected CI [0.487, 2.178], *P* < 0.01). Furthermore, the analysis revealed significant indirect effects in three pathways. The pathway of peer relationships→learning motivation→learning engagement→academic achievement had an indirect effect of 0.191 (t = 2.653, 95% bias-corrected CI [0.076, 0.365], *P* < 0.01). The pathway of peer relationships→learning motivation→learning engagement had an indirect effect of 0.713 (t = 2.493,95% bias-corrected CI [0.193, 1.326], *P* < 0.01). Lastly, the pathway of peer relationships→learning engagement→academic achievement had an indirect effect of 0.293 (t = 2.307, 95% bias-corrected CI [0.081, 0.585], *P* < 0.01). These results indicate that the three mediating effects were all statistically significant, providing support for H4, H8, and H9.

**Fig. 2:**
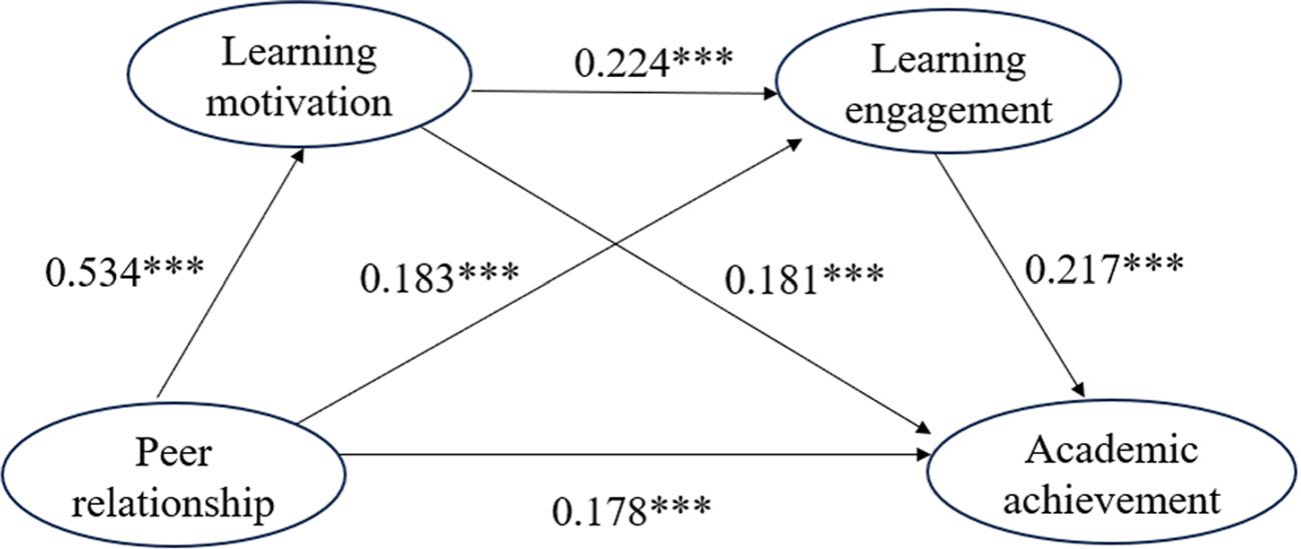
The path diagram, ***p < *0.001*

In addition, the indirect effect percentage of learning motivation and learning engagement as partial mediators were examined. As indicated in Table 6, among the three significant indirect mediators, the indirect effect of learning motivation accounts for 59.5% of the total indirect effect, while the indirect effect of learning engagement accounts for 24.5% of the total indirect effect. Besides, the indirect effect of earning motivation and learning engagement accounts for 16% of the total indirect effect. The pathway “peer relationships → learning motivation → academic achievement” exhibited the strongest effect. The specific pathways of peer relationship acting on academic achievement through learning motivation and learning engagement are detailed in Fig. 2.

**Table 6:** Total, direct, and indirect effects of the theoretical model

Path relationship		Point estimate	Product of coefficient		Bootstrapping			
					Bias-corrected 95% CI		Percentile 95% CI	
			SE	t	Lower	upper	lower	upper
**Test of indirect, direct and total effects**								
DistalIE	PR→LM→LE→AA	0.191	0.072	2.653	0.076	0.365	0.059	0.339
LMIE	PR→LM→AA	0.713	0.286	2.493	0.193	1.326	0.191	1.321
LEIE	PR→LE→AA	0.293	0.127	2.307	0.081	0.585	0.076	0.584
TIE	Total indirect effect	1.198	0.312	3.840	0.656	1.907	0.635	1.870
DE	PR→AA	1.313	0.354	3.712	0.487	2.178	0.496	2.179
TE	total effect	2.510	0.404	6.213	1.745	3.309	1.740	3.290
**Percentage of indirect effects**								
P1	DistalIE/TIE	0.160	0.078	2.051	0.057	0.394	0.051	0.344
P2	LMIE/TIE	0.595	0.137	4.343	0.257	0.787	0.258	0.789
P3	LEIE/TIE	0.245	0.112	2.188	0.065	0.495	0.076	0.502
P4	TIE/TE	0.477	0.128	3.727	0.256	0.777	0.256	0.777
P5	DE/TE	0.523	0.128	4.086	0.223	0.744	0.223	0.744

*Note* PR = Peer Relationship, LM = Learning Motivation, LE = Learning Engagement, AA = academic achievement, IE = Indirect effect, TIE = Total Indirect Effect, DE = Direct Effect, TE = Total Effect, DIE = Distal Indirect Effect

## Discussion

This study aimed to examine the interactive effects of peer relationships, learning motivation, learning engagement, and academic achievement among junior high school students. Additionally, the study sought to investigate the potential mediating roles of learning motivation and learning engagement in the association between peer relationships and academic achievement within this specific context. The study tentatively demonstrated the applicability of SSMMD in explaining the factors influencing academic achievement in junior high school settings. The findings of the study are presented below.

The results of the study revealed a direct and positive association between peer relationships and academic achievement among junior high school students. This finding not only confirms the research result of Jacobson and Burdsal [27], and that of Gallardo et al. [11], showing a positive correlation between peer relationships and academic achievement among middle school students but also reflects the idea presented by Escalante et al. [69] that academic achievement is affected by school climate, of which peer relationships are the dominant factor. This finding can be attributed to the notion that junior high school students in China who have stronger peer relationships within their school environment may receive greater support in their learning endeavors. This increased support may help alleviate learning-related stress, bolster their confidence levels, and enhance their self-esteem, thereby contributing to improved academic performance [26]. Additionally, it is noteworthy that peer influence exerts a substantial impact on shaping students’ academic behavior. For instance, students may observe their peers’ self-regulated behavior and diligence and be inclined to imitate them, thereby adopting similar study habits and strategies [70]. This study further demonstrates that peer relationships are a predictive factor of academic achievement.

The results of the study indicated that learning motivation partially mediated the association between peer relationships and academic achievement among Chinese middle school students. The finding builds upon previous research conducted by Wentzel [17], as it further elucidates the mediating role of learning motivation as a mediator between peer relationships and academic achievement among junior high school students. This finding can be explained by the increased reliance on peers for support and guidance, particularly after transitioning to junior high school. In Chinese culture, where collective values and social harmony are emphasized, peer relationships serve as a crucial source of support and guidance for students [71]. This heightened interaction with peers positively influences their learning attitude and personal values [72]. Consequently, this positive influence on learning attitudes and personal values contributes to the enhancement of learning motivation, ultimately leading to improved academic achievements among junior high school students. Additionally, the study’s results indicated the most substantial mediating role of learning motivation, supporting the notion that motivation is a more critical contributor to academic achievement [25]. This finding provides further evidence of the significant role of learning motivation in mediating the correlation between peer relationships and junior high school students’ academic achievement.

The results of the study demonstrated that learning engagement also partially mediated the association between peer relationships and academic achievement among junior high school students. This suggests that a high level of learning engagement can help elucidate why junior high school students who foster positive relationships with their peers tend to exhibit improved academic performance. When students have positive peer relationships, their increased learning engagement is reflected in their active participation in class, eagerness to complete assignments, and proactive pursuit of additional learning opportunities, ultimately leading to enhanced academic achievement [19]. This finding aligns with prior research [73, 74], which postulates that learning engagement is a pivotal factor linking peer relationships and junior high school students’ academic achievement. The connections that teenagers forge with their contemporaries will facilitate increased participation in the educational process, which in turn will lead to enhanced academic performance [75]. The finding provided more evidence that learning engagement plays a significant role in the link between peer relationships and academic achievement.

The study further revealed that learning motivation and learning engagement played a chain mediation role in the association between peer relationships and academic achievement, which is one of the most astonishing conclusions drawn from the investigation. This result aligns with the self-system model of motivational development [20], which suggests that positive interactions and support from peers contribute to the development of individuals’ learning motivation. This motivation, in turn, influences their level of learning engagement, leading to improved academic achievement. Furthermore, the study revealed that junior high school students’ learning motivation contributed less to their level of learning engagement (β = 0.244, *P* < 0.001) than their peer relationships (β = 0.183, *P* < 0.001). This suggests that junior high school students’ primary source of learning engagement was learning motivation, because motivation plays a crucial role in driving their interest, effort, and persistence in academic tasks [49].

### The theoretical and practical implications

This study holds significant theoretical implications. Firstly, it un derscores the complex interplay between peer relationships, learning motivation, learning engagement, and academic achievement. This expands our understanding of the underlying mechanisms that link these variables together. Secondly, it provides empirical support for the self-system model of motivational development, which suggests that peer relationships have an indirect influence on academic achievement through the mediating roles of learning motivation and learning engagement. This highlights the significance of social factors in shaping students’ motivation and engagement in the learning process.

This study carries practical implications for educators. Firstly, fostering positive peer relationships should be prioritized in educational settings. Teachers should implement strategies to promote a supportive and external classroom environment, such as peer mentoring programs or cooperative learning activities. Besides, teachers should create an inclusive and internal classroom environment that values diversity and promotes respect, empathy, and cooperation. By enhancing positive interactions among students, the motivation and engagement of individuals can be positively influenced, leading to improved academic achievement. Secondly, interventions targeting learning motivation and learning engagement should be implemented. Regarding learning motivation, teachers should encourage students to participate in problem-solving activities that connect learning to students’ lives and experiences, and motivate students to embrace challenges and solve problems [76]. Furthermore, teachers should provide timely and constructive feedback that helps students monitor their learning progress and adjust their strategies accordingly to foster students’ sense of intrinsic motivation. Additionally, teachers should understand the pressures students face in the learning process and provide appropriate support and strategies, such as offering flexible deadlines and providing alternative assignments. To enhance learning engagement, teachers should strive to gain a deeper understanding of teenagers’ needs and employ tactics and skills that strengthen their commitment to learning through meaningful classroom activities. Additionally, emotional support should be provided to help prevent learning fatigue and promote a positive attitude toward the learning process.

This study contributes to the literature in two ways. Firstly, it investigates the complex relationships among peer relationships, learning motivation, learning engagement, and academic achievement utilizing the self-system model of motivational development, which may provide insights for future research in other countries. Secondly, it explores the mediating mechanism between peer relationships and junior high school students’ academic achievement through examining the roles of learning motivation and learning engagement. The novel perspective can enrich our understanding of the link between peer relationships and academic achievement among junior high school students.

### Limitations and future research directions

There are some limitations that should be acknowledged. Firstly, the study was carried out in a cross-sectional manner, making it difficult to establish a causal relationship between variables. Therefore, future longitudinal research is needed to investigate the association between peer relationships and academic achievement more conclusively. Secondly, this study was conducted within the context of China’s test-oriented learning environment, which may limit the generalizability of the findings to other educational settings. To enhance the external validity of the study, future research should be conducted in different countries. Thirdly, the study did not account for potential confounding factors such as academic pressure and self-evaluation, which may also influence academic achievement. Future research should consider these factors within a comprehensive theoretical framework. Finally, apart from academic achievement, all other variables were self-reported by participants, which may introduce potential bias. Future studies could benefit from incorporating observational data from parents, teachers, and classmates to provide a more objective perspective.

## Data Availability

The datasets generated and/or analysed during the current study are not publicly available due to ethical issues but are available from the corresponding author on reasonable request.
